# Application of the “oral simulated tube feeding method” in enteral nutrition for patients with moderately severe acute pancreatitis: a randomized controlled non-inferiority trial comparing the nasogastric feeding route

**DOI:** 10.3389/fnut.2026.1838503

**Published:** 2026-07-31

**Authors:** Sainan Shi, Dan Yang, Yingxin Gao, Zhen Liu, Xinjuan Liu, Huaizhu Guo

**Affiliations:** Department of Gastroenterology, Beijing Chaoyang Hospital, Capital Medical University, Beijing, China

**Keywords:** enteral nutrition, gastroenterology, health care costs, pancreatitis, patient safety

## Abstract

**Objective:**

To evaluate the efficacy and safety of an “oral simulated tube feeding method” compared to nasogastric feeding in patients with moderately severe acute pancreatitis (MSAP).

**Methods:**

In this randomized controlled non-inferiority trial, 60 MSAP patients were assigned to either the oral simulated tube feeding group (STREAM, *n* = 31) or the nasogastric group (NG, *n* = 29). The primary outcome was the 24-h enteral nutrition (EN) tolerance rate.

**Results:**

The 24-h tolerance rate in the STREAM group was non-inferior to the NG group (96.8% vs. 69.0%, *P* < 0.001). Secondary outcomes showed the STREAM group had lower 24-h tolerance scores (1.94 ± 0.26 vs. 3.59 ± 0.34, *P* < 0.001) and fewer occurrences of abdominal distension (*P* < 0.001) and nausea (*P* = 0.025). Additionally, the STREAM group had significantly lower hospitalization costs (*P* = 0.011), with only 12.9% of patients exceeding 10,000 ¥ compared to 48.3% in the NG group. No significant difference was observed in the length of hospital stay.

**Conclusion:**

The oral simulated tube feeding method is a non-inferior, safe, and cost-effective alternative to nasogastric feeding for MSAP patients.

## 1 Introduction

Acute pancreatitis, a sudden inflammatory disorder of the pancreas primarily driven by enzymatic autodigestion, is characterized by persistent systemic inflammatory response and local pancreatic complications, often accompanied by significant hypermetabolism and impaired intestinal barrier function (1, 2). This state not only leads to rapid depletion of nutritional substrates but also, due to increased intestinal permeability and bacterial translocation, significantly elevates the risk of enterogenic infections, directly impacting patient prognosis (3, 4). Therefore, early implementation of effective enteral nutrition (EN) support has become a cornerstone of comprehensive AP management. International authoritative guidelines consistently recommend initiating enteral nutrition (EN) within 24–48 h after the patient’s hemodynamic stability, which helps maintain intestinal mucosal integrity, regulate immune-inflammatory responses, and reduce the incidence of infectious complications and mortality (5–8).

Currently, nasogastric tube feeding (including nasogastric and nasojejunal tubes) is widely recommended by domestic and international guidelines due to its reliable delivery route (9, 10). However, this invasive procedure itself has significant limitations: 10–30% of patients experience nasopharyngeal mucosal injury, pain, or ulceration (11, 12); furthermore, unplanned extubation rates range from 1.5 to 45%, and common discomforts such as pharyngeal foreign body sensation, regurgitation, and restricted mobility severely compromise patient tolerance and compliance (13, 14). These issues not only hinder the smooth implementation of EN and timely achievement of nutritional goals but also increase the medical burden due to managing related complications and potentially prolonged hospital stays.

Oral feeding, as the most physiological EN route, offers the advantages of being non-invasive and naturally stimulating digestive secretion, gastrointestinal motility, and maintaining microbial homeostasis. It has been proven safe and effective in patients with mild acute pancreatitis, promoting recovery (15, 16). However, directly applying this model to patients with moderately severe acute pancreatitis (MSAP), who experience more intense metabolic stress and more severe gastrointestinal dysfunction, currently lacks high-quality evidence support. The core reason is that the unregulated volume and rate of conventional oral intake often trigger a “bolus effect,” leading to rapid gastric distension and overstimulation of the pancreas, which MSAP patients cannot tolerate. Furthermore, there remains a significant research gap: current clinical practice lacks standardized, operable protocols and systematic tolerance assessment systems tailored for the complex gastrointestinal needs of this specific population (17).

To address this clinical challenge, this study proposes an innovative “oral simulated tube feeding method.” Unlike the invasive traditional nasogastric route or the inconsistent delivery of conventional oral feeding, this method involves designing a timed, quantitative, and progressively increasing oral short-peptide enteral nutrition formula regimen to mimic the constant, continuous infusion mode of a nasogastric pump. The core principle lies in achieving sustained and stable intestinal stimulation and nutrient supply through extremely low-dose, high-frequency oral administration, while avoiding excessive one-time intestinal load. This approach balances the non-invasiveness and comfort of the oral route with the dose controllability and continuous infusion of the tube feeding route.

Therefore, this study aims to systematically evaluate the efficacy and safety of the “oral simulated tube feeding method” compared to the traditional nasogastric route in MSAP patients through a prospective, randomized controlled, non-inferiority clinical trial. We hypothesized that the oral simulated tube feeding method would be non-inferior to nasogastric feeding in terms of 24-h enteral nutrition tolerance. Additionally, we hypothesized that this approach would reduce gastrointestinal symptoms and lower hospitalization costs due to its non-invasive nature and improved patient compliance.

## 2 Materials and methods

### 2.1 Study design

This study is a small-sample, open-label (indicating no blinding), prospective, two-arm randomized controlled non-inferiority clinical trial. All participants provided informed consent after understanding the study details. The study was reviewed and approved by the Ethics Committee of Beijing Chaoyang Hospital, Capital Medical University (Ethics Approval Number: 2025-14-42).

### 2.2 Study subjects and inclusion/exclusion criteria

A total of 60 patients with moderately severe acute pancreatitis (MSAP) hospitalized in the Department of Gastroenterology, Beijing Chaoyang Hospital, Capital Medical University, from January 2025 to August 2025 were enrolled.

Inclusion criteria were: ➀Age 18–80 years; ➁Pancreatitis patients classified as MSAP according to the revised Atlanta Classification 2012 (RAC) (18), i.e., AP associated with transient organ failure (≤48 h) or local or systemic complications; ➂ Agreement to receive early enteral nutrition and good compliance; ➃ Voluntary signing of the informed consent form.

Exclusion criteria were:

➀ Severe cardiac, hepatic, pulmonary, renal, hematological, endocrine, or neurological diseases, mental disorders, severe infections in the respiratory, gastrointestinal, or urinary tracts; ➁ Previous history of pancreatic or gastrointestinal surgery (e.g., gastrectomy, intestinal resection, or pancreaticoduodenectomy); ➂ Pregnant or breastfeeding women.

### 2.3 Sample size calculation, randomization, and blinding

To determine that the 24-h enteral nutrition tolerance rate in the experimental group (oral simulated tube feeding group) is non-inferior to the control group (nasogastric feeding group), a non-inferiority test was used. Based on preliminary data (19), the 24-h EN tolerance rate was assumed to be 75% in the experimental group and 53% in the control group. The non-inferiority margin was set at 15%, with α = 0.025 and β = 0.20, and a 1:1 allocation ratio between groups. Using PASS software, 26 patients were required per group. Considering a 10% dropout rate, the sample size was expanded to 29 patients per group, totaling 58 patients. Simple randomization was performed using SPSS 26.0 software to generate random numbers, creating a random allocation sequence with a 1:1 allocation probability. Because simple (unrestricted) randomization was used rather than block randomization, the final group assignment of the 60 enrolled patients naturally resulted in 31 patients in the oral group and 29 in the nasogastric group, which is consistent with the characteristics of this randomization method. To ensure allocation concealment, the allocation sequence was placed in sequentially numbered, opaque, sealed envelopes, which were kept by a third party and opened only after the participant was enrolled. When a subject met the inclusion criteria, the responsible nurse opened the envelope to determine the group assignment and provided corresponding EN education and performed the procedure according to the allocation sequence. Due to the nature of the nutrition delivery methods, blinding of patients and the clinical staff was not feasible; however, the outcome assessors and statisticians were blinded to the treatment allocation throughout the study to minimize detection and reporting bias.

### 2.4 Interventions

According to the 2020 European Society for Clinical Nutrition and Metabolism (ESPEN) clinical nutrition guidelines (20) and the 2021 Chinese guidelines for acute pancreatitis (21): Energy requirements can be estimated based on resting energy expenditure (REE) calculated as 18–20 kcal⋅kg^–1^⋅d^–1^ (based on actual body weight), plus activity or stress factors to estimate individual total energy expenditure (TEE). Protein intake should be 1.2–1.5 g⋅kg^–1^⋅d^–1^. Target requirements should be achieved within 4–7 days. It is important to emphasize that both the experimental and control groups followed identical daily caloric and protein targets for the entire duration of the intervention to ensure rigorous comparability.

Subjects were randomly assigned to two groups:

*Control group (nasogastric feeding):* Enteral nutrition formula was administered via a nutrition pump. A total of 500 mL warm water was thoroughly mixed with 125 g short-peptide enteral nutrition formula peptisorb, Nutricia Company; Each 125g of powder provides 502.5 kcal of energy, 18.38g of hydrolyzed whey protein, 8.38g of fat (including 15% medium-chain triglycerides), and 88.75g of carbohydrates. Subsequently, the amount could be doubled every 24 h. The specific implementation method was:

Day 1: Continuous infusion of short-peptide enteral nutrition formula using a nutrition pump at 50 ml⋅h^–1^ for a total of 12 h.

Day 2: Continuous infusion of short-peptide enteral nutrition formula using a nutrition pump at 100 ml⋅h^–1^ for a total of 12 h.

Day 3: Continuous infusion of short-peptide enteral nutrition formula using a nutrition pump at 150 ml⋅h^–1^ for a total of 12 h.

Day 4: Achieve the target amount of 500 g short-peptide enteral nutrition formula per day, maintaining the same administration method.

*Experimental group (oral simulated tube feeding)*: Enteral nutrition formula was ingested orally at a constant, slow rate. A graduated measuring cup and a 5 mL measuring spoon could be used as tools for EN intake. For EN support, patients needed to slowly and evenly ingest 12.5 g of the same short-peptide enteral nutrition formula mixed with 50 mL warm water (approximately 38–40°C) per hour. Subsequently, the amount could be doubled every 24 h. During the period of oral EN administration, patients still required close monitoring. Patients were instructed to self-manage the ingestion process using the provided measuring tools. Nursing staff explicitly supervised the ingestion process by providing hourly reminders rather than direct administration, and documented the actual ingested volume in a bedside fluid log at each hourly interval to ensure all scheduled intake was successfully completed and adherence was maintained. The high-frequency pacing (e.g., 2 scoops every 10 min) within each hour was executed entirely by the patients independently after initial training, thereby avoiding continuous nursing line interruption. If patients experienced EN intolerance such as abdominal distension, abdominal pain, diarrhea, nausea, or vomiting, symptomatic treatment was required.

Specific implementation method:

Day 1: Prepare 12.5 g short-peptide formula + 50 mL warm water per hour. Orally administer slowly and evenly at a rate of approximately 2 scoops/10 min. Continue oral administration for 12 consecutive hours. Total volume = 600 mL, total energy = 600 kcal⋅d^–1^, total protein = 22.2 g⋅d^–1^.

Day 2: Prepare 25 g short-peptide formula + 100 mL warm water per hour. Orally administer slowly and evenly at a rate of approximately 4 scoops/12 min. Continue oral administration for 12 consecutive hours. Total volume = 1,200 mL, total energy = 1,200 kcal⋅d^–1^, total protein = 44.4 g⋅d^–1^.

Day 3: Prepare 37.5 g short-peptide formula + 150 mL warm water per hour. Orally administer slowly and evenly at a rate of approximately 6 scoops/12 min. Continue oral administration for 12 consecutive hours. Total volume = 1,800 mL, total energy = 1,800 kcal⋅d^–1^, total protein = 66.6 g⋅d^–1^.

Day 4: Achieve the target amount of 500 g short-peptide enteral nutrition formula per day, maintaining the same administration method.

*Basic treatment:* For MSAP patients in both groups, routine interventions were strictly standardized according to the 2021 Chinese guidelines for acute pancreatitis to ensure consistency. These included controlled fluid resuscitation (initial infusion of isotonic crystalloids at 5–10 mL/kg/h to maintain mean arterial pressure > 65 mmHg and urine output > 0.5 mL/kg/h), analgesia (stepwise use of non-steroidal anti-inflammatory drugs or pethidine, with morphine avoided to prevent sphincter of Oddi contraction), nutritional support, and management of early complications (including the restricted use of prophylactic antibiotics and a “step-up” approach for local collections) were provided after hospital admission.

Subjects’ tolerance needed close monitoring during EN. If patients experienced EN intolerance such as abdominal distension, abdominal pain, diarrhea, nausea, or vomiting, the physician had to be notified immediately for symptomatic treatment. Patients with severe adverse reactions, such as paralytic ileus, were withdrawn from the study.

### 2.5 Data collection

Comprehensive data collection was conducted for all enrolled patients at predefined time points. Baseline information was collected upon admission, prior to the initiation of enteral nutrition (EN). This included demographic characteristics [gender, age, body mass index (BMI), and education level], pancreatitis etiology, and baseline disease severity assessments (Ranson score, CTSI score, and Pain score). Baseline inflammatory markers (WBC, Neutrophil %, CRP) were also recorded at this time. Specifically, the Ranson score (range 0–11) was calculated using early clinical and laboratory parameters to evaluate the severity of acute pancreatitis. The CT Severity Index (CTSI, range 0–10) was utilized to assess the extent of pancreatic and peripancreatic inflammation and necrosis based on contrast-enhanced computed tomography findings. Additionally, baseline abdominal pain was quantified using a standard 10-point Numerical Rating Scale (Pain score, range 0–10, where 0 represents no pain and 10 represents the worst imaginable pain).

The scale assesses three main dimensions: (1) Abdominal pain or distension (0: none, 1: mild, 2: moderate/obvious, 3: severe/tense); (2) Nausea or vomiting (0: none, 1: nausea, 2: vomiting 1–2 times/day, 3: persistent vomiting); and (3) Diarrhea (0: none, 1: 3–5 times/day or loose stools, 2: > 5 times/day or watery stools). The total score is the sum of these three dimensions (range 0–9). According to the consensus, a total score ≥ 5 is defined as enteral nutrition intolerance, indicating a high risk of gastrointestinal dysfunction that requires clinical intervention or feeding rate adjustment.

Blood tests were collected upon admission and after feeding. For laboratory analyses, including baseline inflammatory markers (WBC, Neutrophil %, CRP) and nutritional outcome indicators (serum Albumin and Prealbumin), approximately 3–5 mL of fasting venous blood was collected from patients after at least an 8-h fast. The blood samples were promptly centrifuged, and the serum levels of these indicators were detected using an automated biochemical analyzer (e.g., Beckman Coulter AU5800) via standard colorimetric and immunoturbidimetric methods.

Safety outcomes were also monitored, including nasogastric-related complications (nasopharyngeal mucosal injury and unplanned extubation) and serious adverse events (SAEs) such as paralytic ileus. Nasopharyngeal mucosal injury was defined as clinical evidence of localized pain, ulceration, or bleeding in the nasal or pharyngeal cavity following tube insertion.

*Primary outcome:* 24-h enteral nutrition tolerance, defined as the proportion of patients with an EN tolerance score < 5.

*Secondary outcomes:* 72-h enteral nutrition tolerance, improvement in nutritional laboratory parameters, specifically serum Albumin (g/L) and Prealbumin (g/L), hospital length of stay, and hospitalization costs, and the incidence of adverse reactions (including nasogastric-related complications and serious adverse events.

Outcome measures were strictly evaluated according to the study protocol timeline: (1) EN tolerance scores and gastrointestinal symptoms were assessed at exactly 24 and 72 h following the initiation of the nutritional intervention. To minimize reporting bias regarding subjective symptoms (such as nausea and distension) in this open-label setting, all symptom evaluations and score calculations were performed by independent outcome assessors who were strictly blinded to the patients’ group allocations, utilizing the standardized 10-point scale; (2) Nutritional laboratory indicators (serum Albumin and Prealbumin) were collected before the start of EN and subsequently after the completion of the EN support period to evaluate nutritional improvement; (3) Data regarding the length of hospital stay, total hospitalization costs, and the occurrence of any EN-related complications or serious adverse events were monitored continuously and recorded upon patient discharge.

### 2.6 Outcome measures

The enteral nutrition tolerance assessment scale [based on the 2021 Expert Consensus on Enteral Nutrition for Gastrointestinal Dysfunction in Critically Ill Patients (22)] was used for evaluation. Prior clinical validations of this tool have demonstrated good internal consistency, with a Cronbach’sα coefficient of 0.85, indicating high reliability in assessing gastrointestinal symptoms. Furthermore, the scale exhibits strong content validity, as its dimensions and scoring thresholds were strictly established and validated through the Delphi method by the critical care expert consensus panel.

### 2.7 Statistical analysis

Non-inferiority and superiority Z-tests for the primary outcome were performed using SAS 9.4.1. Efficacy outcomes, including tolerance rates, were evaluated using a per-protocol (PP) analysis, meaning patients who were withdrawn due to severe adverse events prior to specific time points were excluded from that respective time point’s denominator. Statistical analysis was conducted using SPSS 26.0 software. Continuous variables with normal distribution were expressed as mean ± standard deviation (±s ) and compared using independent samples *t*-tests. The normality of continuous variables was assessed using the Shapiro-Wilk test. Continuous variables with non-normal distribution were expressed as median (M) and interquartile range (P_25_, P_75_) and compared using the Mann-Whitney U test. Effect size indicators and 95% confidence intervals (CI) were calculated for all major outcomes. For the primary outcome, non-inferiority was established if the lower limit of the two-sided 95% CI for the difference in tolerance rates (Experimental minus Control) was greater than the non-inferiority margin of -15%. For continuous clinical outcomes, the mean difference (MD) and its 95% CI were reported.

Variables with P < 0.05 in univariate analysis were included in multivariate logistic regression analysis. The goodness-of-fit of the logistic regression model was evaluated using the Hosmer-Lemeshow test and the Akaike Information Criterion (AIC). Additionally, multicollinearity among the independent variables was assessed using the Variance Inflation Factor (VIF), with a VIF < 5 considered to indicate the absence of significant multicollinearity. A *P* < 0.05 was considered statistically significant.

## 3 Results

### 3.1 Comparison of general data

A total of 60 patients with moderately severe acute pancreatitis (MSAP) were included in this study, with 31 patients in the oral simulated tube feeding group and 29 patients in the nasogastric feeding group. There were no statistically significant differences between the two groups in terms of gender, age, education level, and BMI, and pancreatitis etiology (including hyperlipidemic, biliary, and alcoholic causes) (*P* > 0.05, see Table 1).

**TABLE 1:** Comparison of baseline characteristics and enteral nutrition initiation time between the two groups.

Characteristic	Oral simulated tube feeding (*N* = 31)	Nasogastric feeding (*N* = 29)	*P*-value
Demographics and general data			
Gender (n, %)			0.464
Male	23 (74.2)	19 (65.5)	
Female	8 (25.8)	10 (34.5)	
BMI (kg/m^2^)	28.02 ± 0.829	26.84 ± 0.872	0.329
Age (years) (n, %)			0.325
18–25	3 (9.7)	2 (6.9)	
26–30	3 (9.7)	2 (6.9)	
31–40	11 (35.5)	7 (24.1)	
41–50	5 (16.1)	7 (24.1)	
51–60	3 (9.7)	5 (17.2)	
> 60	6 (19.4)	6 (20.7)	
Etiology (n, %)			0.912
Hyperlipidemic	12 (38.7)	10 (34.5)	
Biliary	11 (35.5)	12 (41.4)	
Alcoholic	5 (16.1)	4 (13.8)	
Other	3 (9.7)	3 (10.3)	
Education level (n, %)			0.778
Middle school or below	6 (19.4)	7 (24.1)	
High school	8 (25.8)	7 (24.1)	
College/undergraduate	14 (45.2)	12 (41.4)	
Postgraduate or above	3 (9.7)	3 (10.3)	
Baseline laboratory indicators			
WBC (×10^9^/L)	12.58 ± 0.528	12.10 ± 0.568	0.248
Neutrophil %	84.12 ± 1.017	83.45 ± 1.062	0.651
CRP (mg/L)	90.35 ± 10.06	99.66 ± 13.16	0.575
Albumin (g/L)	43.33 ± 2.089	40.22 ± 1.372	0.225
Prealbumin (g/L)	4.820 ± 3.287	3.110 ± 2.078	0.661
Ranson score	4 (3, 4)	4 (3, 4)	0.453
CTSI score	4 (2, 4)	3 (3, 4)	0.665
Pain score	2 (2, 2)	2 (2, 2)	0.666
Intervention details			
EN initiation time (h)			0.022
<24	2 (6.5)	1 (3.4)	
24–48	12 (38.7)	21 (72.4)	
48–72	11 (38.5)	7 (24.1)	
>72	6 (19.4)	0 (0)	

### 3.2 Comparison of baseline clinical data

There were no statistically significant differences between the oral simulated tube feeding group and the nasogastric feeding group regarding inflammation-related indicators, nutritional indicators, and disease severity before EN support (*P* > 0.05, see Table 1).

### 3.3 Comparison of enteral nutrition initiation time

There was a statistically significant difference in the time to initiate EN between the oral simulated tube feeding group and the nasogastric feeding group (*P* < 0.05). In the oral group, 6 patients had EN initiated > 72 h after admission (see Table 1).

### 3.4 Comparison of primary clinical outcomes

The 24-h EN tolerance rates in the oral simulated tube feeding group and nasogastric feeding group were 96.8 and 69.0%, respectively. The rate difference between the two groups was 27.8% (95% CI: 9.8–45.8%). The non-inferiority *Z*-test confirmed that the oral group was non-inferior to the nasogastric group (*Z* = 4.674, *P* < 0.001), as the lower limit of the 95% CI (9.8%) was well above the pre-specified non-inferiority margin of -15%. Furthermore, because the entire 95% CI for the rate difference (9.8–45.8%) lies well above zero, the data strongly suggests a potential superiority of the STREAM method in achieving 24-h tolerance, although the formal superiority Z-test was marginally insignificant (*P* = 0.081). Additionally, for the 24-h tolerance score, the mean difference between groups was -1.65 (95% CI: -2.48 to -0.82, *P* < 0.001), indicating a reduction in the oral group. Further superiority *Z*-test did not achieve superiority (*Z* = 1.399, *P* = 0.081). The 72-h EN tolerance rate was 100% in both groups (based on the per-protocol analysis, where the single patient in the nasogastric group withdrawn due to paralytic ileus was excluded from the 72-h assessment). Compared to the nasogastric group, the oral simulated tube feeding group had a lower 24-h tolerance score and fewer occurrences of EN intolerance symptoms related to abdominal pain/distension and nausea/vomiting at 24 h of nutritional support (see Table 2).

**TABLE 2:** Comparison of enteral nutrition tolerance between the two groups.

Time point	Outcome measure	Oral simulated tube feeding (*N* = 31)	Nasogastric feeding (*N* = 29)	*P*-value
24 h	EN tolerance (n, %)	30 (96.8)	20 (69.0)	< 0.001
	Tolerance score	1.94 ± 0.262	3.59 ± 0.338	< 0.001
	Abdominal pain/distension	0 (0, 1)	2 (1, 2)	< 0.001
	Nausea/vomiting	1 (0, 1)	1 (1, 2)	0.025
	Diarrhea	0 (0, 1)	1 (0, 1)	0.341
72 h	EN tolerance (n, %)	31 (100)	29 (100)	
	Tolerance score	0.94 ± 0.217	1.52 ± 0.241	0.078
	Abdominal pain/distension	0 (0, 0)	1 (0, 1)	0.007
	Nausea/vomiting	0 (0, 0)	0 (0, 1)	0.074
	Diarrhea	0 (0, 0)	0 (0, 1)	0.236

### 3.5 Comparison of nutritional indicators before and after enteral nutrition

To evaluate the nutritional efficacy of the two feeding methods, we compared serum Albumin and Prealbumin levels before and after the intervention. Results from the two-way repeated measures ANOVA indicated a significant time effect for both Albumin (*F* = 15.42, *P* < 0.001) and Prealbumin (*F* = 21.68, *P* < 0.001), showing that nutritional status improved significantly in both groups following enteral nutrition support. There was no significant interaction between the feeding method and time (*P* > 0.05), suggesting that the “oral simulated tube feeding method” is as effective as the nasogastric route in improving the nutritional markers of MSAP patients (see Table 3).

**TABLE 3:** Changes in nutritional indicators before and after enteral nutrition.

Indicator	Group	Before EN	After EN	Change (Δ)	*P*-value (Time)
Albumin (g/L)	Oral (*N* = 31)	43.33 ± 2.09	45.12 ± 1.85	1.79	< 0.001
	Nasogastric (*N* = 29)	40.22 ± 1.37	41.95 ± 1.54	1.73	
Prealbumin (g/L)	Oral (*N* = 31)	4.82 ± 3.29	6.45 ± 2.15	1.63	< 0.001
	Nasogastric (*N* = 29)	3.11 ± 2.08	4.68 ± 1.92	1.57	

### 3.6 Comparison of secondary outcomes

There was no statistically significant difference in hospital length of stay between the two groups (*P* > 0.05). The median length of stay was 8.0 (7.0, 10.0) days in the oral simulated tube feeding group and 9.0 (8.0, 11.0) days in the nasogastric feeding group (*P* = 0.073). The oral simulated tube feeding group showed a significant advantage in reducing patient hospitalization costs (*P* < 0.05). The median cost in the oral group was 8.2 (7.6, 9.4) thousand ¥, significantly lower than the 10.5 (9.2, 12.1) thousand ¥ observed in the nasogastric group (*P* = 0.011, see Table 4).

**TABLE 4:** Comparison of hospital stay and costs between the two groups.

Outcome	Oral simulated tube feeding (*N* = 31)	Nasogastric feeding (*N* = 29)	*P*-value
Hospital stay (days), *M*(*P*_25_,*P*_75_)	8.0 (7.0,10.0)	9.0 (8.0,11.0)	0.073
Hospital cost (thousand ¥), *M*(*P*_25_,*P*_75_)	8.2 (7.6,9.4)	10.5 (9.2,12.1)	0.011

### 3.7 Analysis of factors influencing enteral nutrition tolerance

The results showed that the EN route was an independent influencing factor for EN tolerance, and oral simulated tube feeding was a protective factor for EN tolerance [OR = 12.252, 95% CI (1.372, 109.387), *P* = 0.025]. The model demonstrated a good fit, as indicated by the Hosmer-Lemeshow test (χ^2^ = 4.321,P = 0.827) and an AIC value of 54.12. Importantly, although there was a baseline difference in EN initiation time between the two groups, the multivariate analysis confirmed that EN initiation time was not an independent influencing factor for 24-h EN tolerance [OR = 1.298, 95% CI (0.329, 5.111), *P* = 0.710]. Furthermore, the VIF values for all independent variables (EN Method, and EN Initiation Time) ranged from 1.08 to 1.45, confirming that there was no significant multicollinearity in the model (see Tables 5, 6).

**TABLE 5:** Assignment table for categorical variables.

Variable	0	1	2	3	4
24 h EN tolerance	Intolerant (Score ≥ 5)	Tolerant (Score < 5)			
EN method Pancreatitis etiology EN initiation time	Nasogastric	Oral			
		Hyperlipidemic	Biliary	Alcoholic	Other
		<24 h	24–48 h	48–72 h	> 72 h

**TABLE 6:** Binary logistic regression analysis of factors influencing EN tolerance.

Variable	β	S.E.	Wald χ ^2^	OR	95% CI	*P*-value	VIF
EN method	2.506	1.117	5.032	12.252	1.372–109.387	0.025	1.08
Pancreatitis etiology	0.044	0.602	0.005	1.045	0.321–3.339	0.941	1.22
EN initiation time	0.261	0.699	0.139	1.298	0.329–5.111	0.710	1.45

### 3.8 Comparison of safety and complications

The oral simulated tube feeding group demonstrated a significant safety advantage regarding procedure-related complications. In the nasogastric feeding group, 13.8% of patients (*n* = 4) experienced nasopharyngeal mucosal injury and 6.9% (*n* = 2) removal of the tube in advance due to intolerance. By design, these complications were completely avoided in the oral group (0%, *P* < 0.05). Regarding serious adverse events, one patient in the nasogastric group (3.4%) developed paralytic ileus and was withdrawn from the study, while no such events occurred in the oral group (see Table 7).

**TABLE 7:** Comparison of complications and safety outcomes between groups.

Outcome measure	Oral simulated tube feeding (*N* = 31)	Nasogastric feeding (*N* = 29)	*P*-value
Nasogastric-related complications			
Nasopharyngeal mucosal injury (n, \%)	0 (0.0)	4 (13.8)	0.048
Unplanned extubation (n, \%)	0 (0.0)	2 (6.9)	0.230
Serious adverse events			
Paralytic ileus (n, \%)	0 (0.0)	1 (3.4)	0.483

## 4 Discussion

This randomized controlled non-inferiority trial is the first to demonstrate that a standardized “oral simulated tube feeding method” designed for MSAP patients is non-inferior to the traditional nasogastric route in achieving early enteral nutrition tolerance. The oral group exhibited a non-inferior tolerance rate at 24 h post-intervention (96.8% vs. 69.0%, *P* < 0.001). Our data specifically showed that patients in the oral group had significantly lower tolerance scores (1.94 ± 0.26vs.3.59 ± 0.34,P < 0.001) and a markedly lower incidence of tube-related abdominal pain and distension. These results directly validate the advantage of a physiological oral route over the invasive nasogastric procedure, which often causes pharyngeal discomfort and restricts patient mobility.

In terms of health economics, the significantly lower median hospitalization cost in the oral group (8.2 vs. 10.5 thousand ¥, *P* = 0.011) represents a tangible clinical advantage. Unlike general literature that broadly mentions “cost-effectiveness” (23), our findings pinpoint the economic benefit derived from the complete elimination of costs associated with disposable nasogastric tubes and nutrition pump sets, as well as the reduction in managing tube-related mucosal injuries. While the STREAM method requires structured hourly nursing reminders, empowering patients to self-manage their intra-hour intake using standardized measuring tools effectively offsets potential increases in labor costs, making the protocol practically feasible in standard clinical wards without creating an unsustainable nursing burden. However, it is noteworthy that there was no statistically significant difference in the length of hospital stay between the two groups (8.0 vs. 9.0 days, *P* = 0.073). We must objectively acknowledge that while the 24-h tolerance metric was significantly improved by the STREAM method, this early advantage did not translate into a statistically significant reduction in the overall length of hospital stay. This suggests that while the “oral simulated tube feeding method” improves immediate comfort and reduces direct medical expenses, the overall duration of hospitalization may be more closely tied to the natural resolution of pancreatic inflammation and the management of local complications rather than the nutrition delivery route alone. Regarding safety and clinical positioning, the STREAM method offers distinct conceptual advantages and boundaries compared to alternatives like nasojejunal (NJ) feeding. While MSAP patients are prone to gastroparesis, the high-frequency, low-volume nature of the STREAM protocol inherently minimizes gastric pooling, thereby reducing the risk of aspiration compared to traditional bolus oral feeding. However, it is fundamentally a gastric delivery method. According to ESPEN guidelines, NJ feeding remains the standard, indicated alternative for patients with profound gastroparesis or persistent intolerance who fail gastric delivery. Therefore, the STREAM method should be conceptualized as an optimal, non-invasive first-line step to maximize gastric tolerance, reserving invasive NJ tube placement as a rescue strategy for refractory cases.

Despite its advantages, the clinical application of this oral protocol has specific limitations that must be objectively considered. Firstly, the “oral simulated tube feeding method” requires the patient to be fully conscious and highly cooperative to maintain the “timed and quantified” intake (e.g., 2 scoops every 10 min). Patients with severe cognitive impairment or those at high risk of aspiration are not suitable candidates for this approach. Secondly, the 24-h EN tolerance rate is a relatively short-term, symptom-based outcome. Because both groups achieved 100% tolerance at 72 h and there was no significant difference in hospital stay, our findings primarily suggest improved early tolerability and patient comfort rather than a clear benefit in more clinically meaningful long-term outcomes. Additionally, our study did not assess long-term outcomes such as the recovery of pancreatic exocrine function, which remains an area for future research. Thirdly, while adequately powered for the primary non-inferiority outcome based on our calculations, the total sample size of 60 patients remains relatively small, warranting future large-scale, multicenter trials to validate these findings.

## 5 Conclusion

In summary, this study indicates that the “oral simulated tube feeding method” designed for MSAP patients is a safe, effective, and economical route for implementing enteral nutrition. This standardized oral protocol is non-inferior to conventional nasogastric feeding, with potential advantages in patient comfort and medical costs. This provides a reliable alternative to invasive tube placement in clinical practice, serving as an optimal physiological alternative specifically for cooperative patients with intact cognitive status. This supports a shift in enteral nutrition models toward a more physiological and patient-centered approach, which has positive clinical significance and promotional value.

## Data Availability

The original contributions presented in this study are included in the article/supplementary material, further inquiries can be directed to the corresponding author.
